# Chronic HCV Infection Affects the NK Cell Phenotype in the Blood More than in the Liver

**DOI:** 10.1371/journal.pone.0105950

**Published:** 2014-08-22

**Authors:** Cormac Cosgrove, Christoph T. Berger, Daniela C. Kroy, Patrick C. Cheney, Musie Ghebremichael, Jasneet Aneja, Michelle Tomlinson, Arthur Y. Kim, Georg M. Lauer, Galit Alter

**Affiliations:** 1 Ragon Institute of MGH, MIT and Harvard, Cambridge, Massachusetts, United States of America; 2 Massachusetts General Hospital, Boston, Massachusetts, United States of America; 3 Medical Department 3, RWTH Aachen University Hospital, Aachen, Germany; 4 Harvard Medical School, Boston, Massachusetts, United States of America; University of Montreal Hospital Research Center (CRCHUM), Canada

## Abstract

Although epidemiological and functional studies have implicated NK cells in protection and early clearance of HCV, the mechanism by which they may contribute to viral control is poorly understood, particularly at the site of infection, the liver. We hypothesized that a unique immunophenotypic/functional NK cell signature exists in the liver that may provide insights into the contribution of NK cells to viral control. Intrahepatic and blood NK cells were profiled from chronically infected HCV-positive and HCV-negative individuals. Baseline expression of activating and inhibitory receptors was assessed, as well as functional responses following stimulation through classic NK cell pathways. Independent of HCV infection, the liver was enriched for the immunoregulatory CD56^bright^ NK cell population, which produced less IFNγ and CD107a but comparable levels of MIP1β, and was immunophenotypically distinct from their blood counterparts. This profile was mostly unaltered in chronic HCV infection, though different expression levels of NKp46 and NKG2D were associated with different grades of fibrosis. In contrast to the liver, chronic HCV infection associated with an enrichment of CD161^low^perforin^high^ NK cells in the blood correlated with increased AST and 2B4 expression. However, the association of relatively discrete changes in the NK cell phenotype in the liver with the fibrosis stage nevertheless suggests an important role for the NK response. Overall these data suggest that tissue localization has a more pervasive effect on NK cells than the presence of chronic viral infection, during which these cells might be mostly attuned to limiting immunopathology. It will be important to characterize NK cells during early HCV infection, when they should have a critical role in limiting infection.

## Introduction

Chronic Hepatitis C virus (HCV) infection is a major health care burden, with 150–170 million people chronically infected worldwide. Traditional treatment protocols are burdened with side-effects and only effective in ∼50% of cases [1], and although effective new medications have recently become available, these are very costly and thus the need for a protective vaccine against HCV remains [2], [3]. However, approaches focused on inducing effective T cell responses [2] or HCV-specific antibodies have had limited success [4], thus research into the stimulation of innate immune effectors as therapeutic or vaccine targets is warranted.

Natural killer (NK) cells are innate immune cells specialized in the elimination of virus-infected cells. They account for up to 15% of peripheral blood lymphocytes and are prevalent at higher frequencies in tissues such as the liver [5]. They can be divided into functionally distinct subsets based on their level of CD56 surface expression: the mainly cytotoxic CD56^dim^ population and the more immunoregulatory cytokine-producing CD56^bright^ NK cell subset. While these subsets account for 90% and 10% of total NK cells in the peripheral blood respectively [6], [7] these relative frequencies are reversed in secondary lymphoid organs including the liver, with the majority of NK cells CD56^bright^. The functions of both NK cell subsets are modulated by inhibitory and activating signals provided through distinct classes of receptors. The best described of these include the killer cell immunoglobulin-like receptors (KIR), the C-type lectins (NKG2), the activating natural cytotoxicity receptors (NCR), and the FcγRIIIa receptor (CD16) that mediates antibody-dependent cytotoxicity [8]. Together, these receptors modulate NK cell function to allow rapid targeting and destruction of virally infected cells without the need for prior sensitization, making them an important first line of defense against viral pathogens.

NK cells may be particularly relevant in the setting of HCV infection, a hepatotropic virus, given that NK cells account for up to 50% of the liver-resident lymphocyte population [9]. Significantly, epidemiological evidence has strongly linked enhanced HCV clearance and sustained viral control with homozygosity of the NK killer cell immunoglobulin-like receptor gene *KIR2DL3* and its ligand HLA-C group 1 alleles [10]–[12]. Moreover, immunophenotypic analyses of peripheral blood NK cells have identified both a distinct NK cell profile in acute infection that predicts HCV clearance [13] and altered NK cell profiles in chronic infection [14]–[16], while more recently, NK cell modulation of virus-specific T cells has been implicated in enhanced viral clearance and reduced immunopathology in mouse models of acute and chronic viral infection [17]. Therefore, changes in specific NK cell subsets may dictate or contribute to disease progression in HCV infection.

Despite the identification of NK cell profiles associated with HCV in the peripheral blood, the key to understanding which cells are most important in antiviral control is likely to lie in the liver, given this is the site of viral replication. Moreover, as intra-hepatic NK cell populations are phenotypically and functionally distinct from those of the peripheral blood, it is likely that unique signatures and functional subsets may contribute to the antiviral response at this target site [18]. As such, the inclusion of HCV-uninfected livers to control for tissue-specific differences is crucial and has been lacking in the majority of studies, which to date have reported conflicting results [15], [16], underlining the uncertainty regarding the effects of HCV infection on liver-resident NK cells in chronic disease. Thus, here we sought to define novel NK cell signatures in HCV-infected livers compared to matched blood samples.

## Materials and Methods

### Subjects, cohort characteristics and sample preparation

Liver specimens were obtained from surgical resection of liver tissue or liver explantation at the Massachusetts General Hospital Gastrointestinal Unit and the Department of Surgery. When intrahepatic lesions were present, the tissue was collected from the most distant section of the specimen. We analyzed 9 HCV-positive liver tissues with paired blood samples, 14 non-HCV diseased liver tissues (six with paired blood samples), and eight blood samples from healthy controls. Clinical characteristics of HCV-infected individuals are described in Table 1, and the reason for liver transplant in the HCV-uninfected individuals are described in Table 2. Intrahepatic lymphocytes were extracted as previously described [19].

**Table 1:** Clinical characteristics of HCV-infected individuals.

Liver Sample	Age	Sex	AST IU/mL^#^	ALT IU/mL^#^	Viral Load IU/ml	Risk factor*	GT*	Ethnicity
Resection	71	M	61	74	5850000	ND	4	White
Resection	56	M	54	59	ND	IVDU	ND	White
Explant	64	M	58	65	1610000	ND	4	African American
Resection	55	F	37	52	ND	ND	ND	White
Explant	61	M	159	65	688000	IVDU	1	White
Resection	64	M	91	108	17900	ND	1	White
Resection	65	M	ND	ND	6820	ND	ND	White
Resection	50	M	86	71	88500	IVDU	ND	White
Explant	46	F	1006	ND	320150	ND	2	White

*p.i. =  post infection; Risk factor for HCV acquisition; GT =  HCV genotype; ND = not determined. # normal range of ALT = 7–56 IU/ml, AST = 5–40 IU/ml.

**Table 2:** Reasons for liver surgery in non-HCV infected individuals.

Number of Liver samples	Reason for surgery
5	CRC metastasis
1	Primary biliary cirrhosis
2	Hepatocellular Carcinoma
1	AIH
1	Adenoma
1	Ileal cancer metastasis
1	Cryptogenic cirrhosis
1	Choledocholithiasis and recurrent cholangitis
1	Oro-pharyngeal cancer metastasis

### Ethics statement

All subjects in this study provided written informed consent prior to sampling. The study was conducted according to the principles expressed in the declaration of Helsinki, and was approved by the Partners Human Research Committee (Title: ‘Cell mediated immunity in Hepatitis C infection’. Protocol #1999-P-004983/54; MGH #90-7246).

### NK cell immunophenotyping

**Figure 1:**
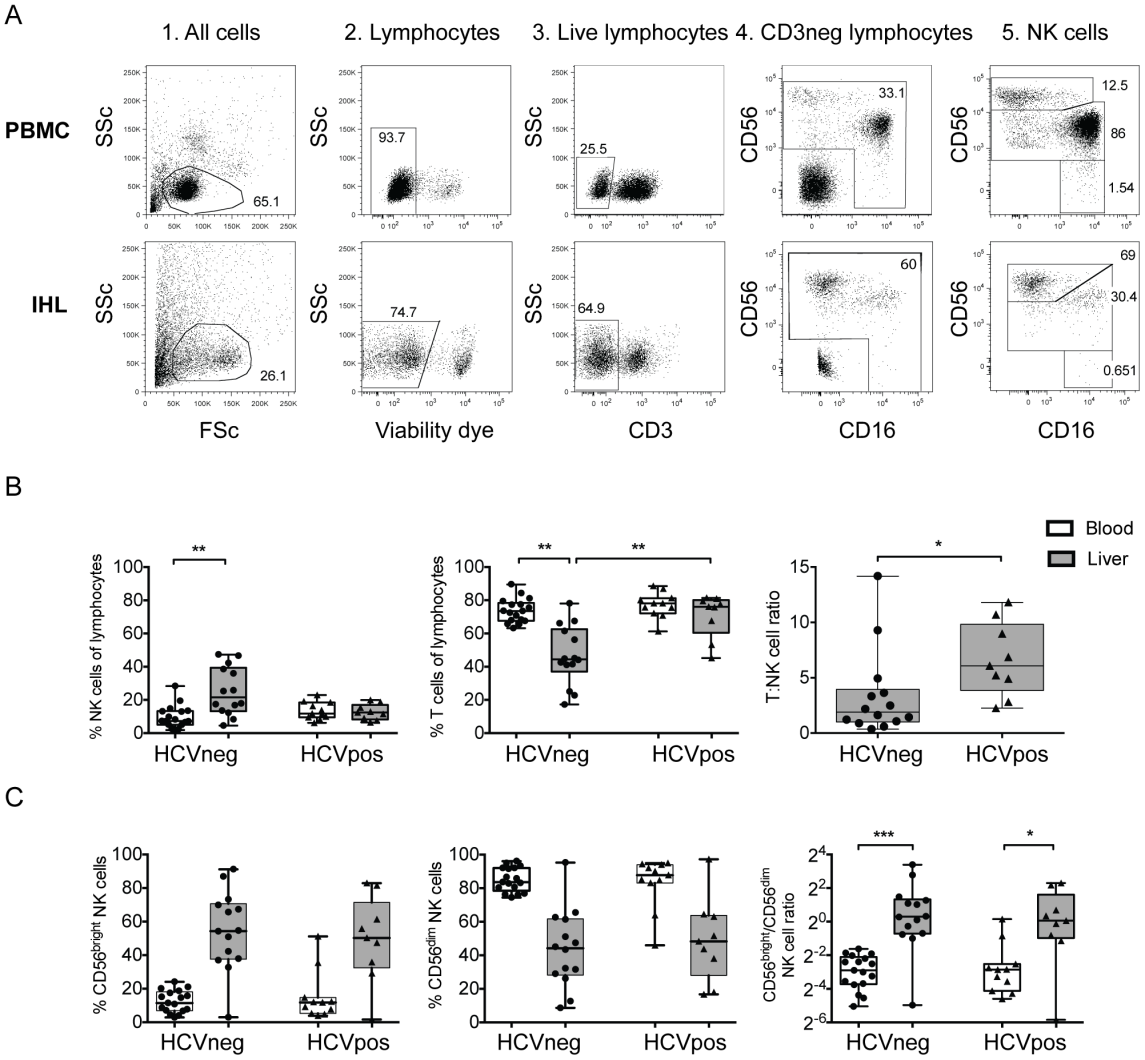
NK cell subsets and distribution in liver and blood. NK cells were identified in the blood and liver of groups of HCV-infected and -uninfected individuals by flow cytometry using a robust gating strategy (A). The size of the NK and T cell populations as a percentage of the total lymphocyte population, as well as the T:NK cell ratio was calculated in the liver and blood in HCV-infected and -uninfected individuals (B), as was the contribution of the CD56^bright^ and CD56^dim^ NK cell subsets to the total NK cell population (C). Statistical significance was accepted at p<0.05 and is indicated by * (p<0.05), ** (p<0.01) and *** (p<0.001).

Flow cytometry was performed on cryopreserved cells. As per the gating strategy in figure 1A, NK cells were identified using a combination of Fixable blue dead cell stain (Life technologies), CD3-Pacific Blue, CD56-PE-Cy7 and CD16- APC-Cy7 (BD Biosciences). NK cell receptor expression was assessed using combinations of CD158a-PerCP-Cy5.5 (eBioscience), CD158b-FITC, KIR3DL1-Alexafluor700 (both Biolegend), KIR2DL3-PE, KIR2DL1-APC (both R&D Systems), NKG2A-PE (Beckman Coulter), NKG2D-APC, CD94-FITC, 2B4-FITC, NKp46-PE, NKp30-APC (all BD Biosciences), TRAIL-PE (Biolegend), CD161-FITC (Miltenyi) and CD160-Alexafluor647 (Biolegend). For intracellular staining, cells were fixed and permeabilized (PermA/B solution, Caltag) according to the manufacturers' instructions, prior to incubation with Perforin-PerCP-Cy5.5 (eBioscience). At least 1500 NK cells were acquired for all samples on either a five laser BD LSRFortessa or a four laser BD LSRII system, equipped with FACSDiva Version 8.8.3 (BD biosciences). Rainbow beads ensured a consistent, comparable level of fluorescence across all samples on different days of acquisition. Gates were set using fluorescence minus one or unstimulated samples where appropriate. The data were analysed using FlowJo version 9.5.3 (Treestar, OR, USA).

### Analysis of NK cell function

NK cell responses to target cell lines were determined as previously described [13]. Briefly, mononuclear cells were incubated with MHC class I-devoid K562, 721.221, or antibody-coated p815 cell lines (ATCC) at an effector to target ratio of 10∶1. CD107a-PECy5 (10 µl/ml), brefeldin A (0.5 µg/ml, Sigma-Aldrich) and monensin (0.5 µg/ml, GolgiStop; BD Biosciences) were added at the start of the incubation. After five hours, cells were washed and stained to identify NK cells as above. Cells were fixed, permeabilized and stained for intracellular cytokines using MIP1β-PE and IFNγ-FITC (both BD Biosciences) antibodies. Multi-parameter flow cytometry was performed on a 4 laser BD LSRII system and analyzed as above.

### Statistical analyses

Descriptive measures (such as frequency, mean, median, standard deviation and interquartile range) were used to summarize data. The Mann-Whitney test was used for the comparison of phenotype and functional data between two groups. For more than two groups comparison, the Kruskal–Wallis test with Dunn's post hoc analyses was used. Spearman's rank correlation was used to examine bivariate associations. All P values are two-sided and P values of <0.05 were considered significant. Statistical analyses were conducted using GraphPad Prism (GraphPad Prism Software, La Jolla, CA). Heatmaps were generated using Genepattern v3.8.0 (Broad Institute, Cambridge, MA) and display colors based on relative values within each phenotypic marker.

## Results

### Intrahepatic lymphocytes are enriched for CD56^bright^ NK cells independent of HCV infection

Similar to previous reports showing an enrichment of NK cells in healthy liver compared to the peripheral blood [20], [21], we observed more NK cells in the liver of uninfected individuals compared to the blood (25.27% vs. 9.8%, p<0.01, Figure 1B). This difference was not seen in the setting of HCV infection, though this has been suggested to be a consequence of relative dilution due to T-cell infiltration [16], [22] which is supported by the increased T∶NK cell ratio in HCV-infected livers (Figure 1B).

Given the altered distribution of the functionally distinct CD56^bright^ and CD56^dim^ NK cell subsets in secondary lymphoid tissues [18] and the liver [23], we next investigated whether these populations were skewed in the context of HCV infection. As described for other tissue-resident NK cells [18], we observed an enrichment of the immunoregulatory CD56^bright^ population over the cytotoxic CD56^dim^ population in the liver versus the blood of both HCV-infected (p = 0.0005) and -uninfected (p<0.0001) individuals (Figure 1C). This enrichment was striking, with CD56^bright^ NK cells accounting for up to 85% of the total NK cell population in the liver of some individuals. However no difference in the subset distribution was observed among infected or uninfected subjects, demonstrating that certain NK cell populations universally compartmentalize to the liver, which remain relatively unperturbed in HCV infection.

### Liver resident CD56^bright^ NK cells display an altered immunophenotype, independent of HCV infection

**Figure 2:**
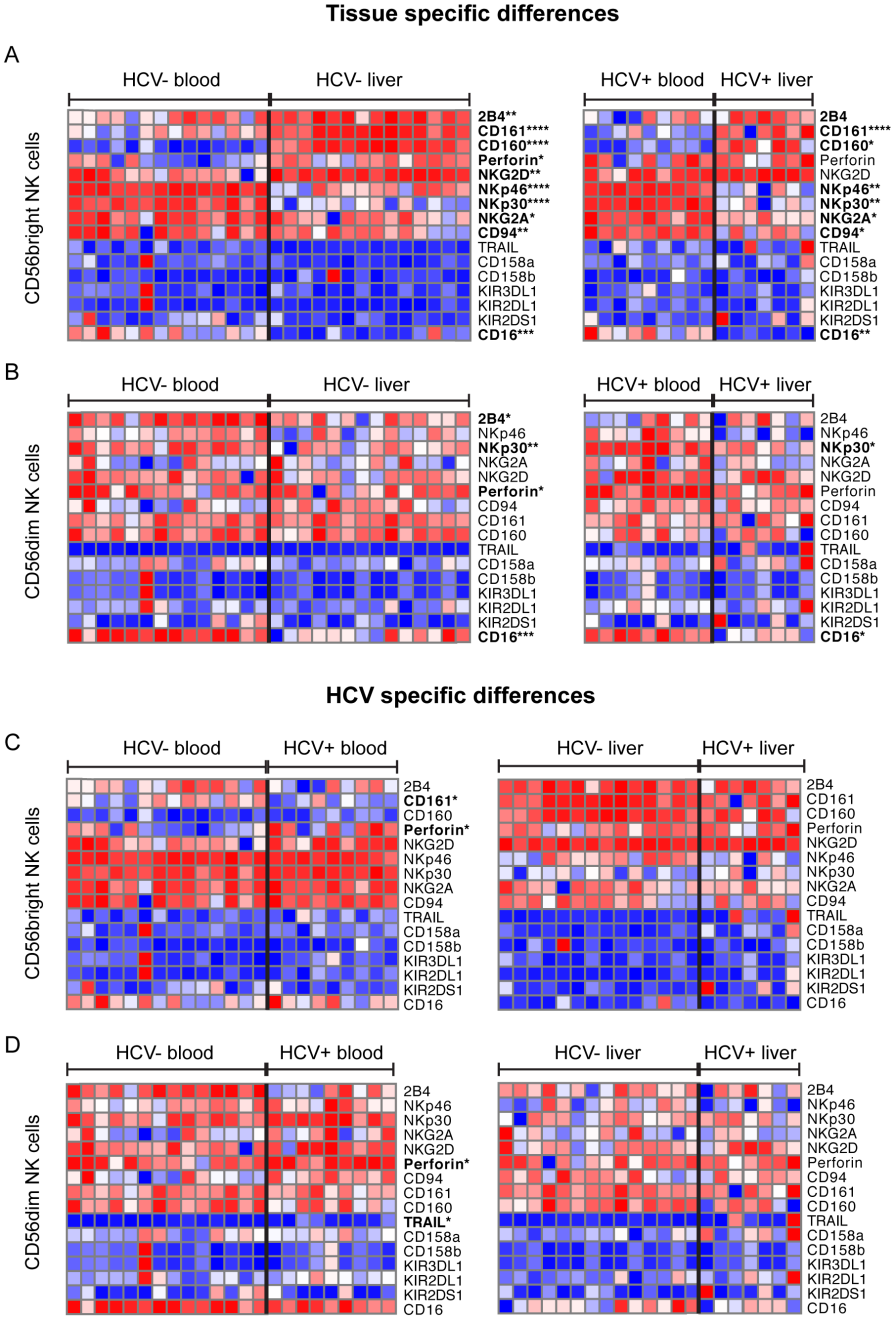
Immunophenotypic profiles of blood and liver-resident NK cell subsets. Immunophenotyping of liver and blood NK cell subsets in groups of HCV-infected and –uninfected individuals was performed by flow cytometry, and both tissue-specific differences (A) and disease-specific differences (B) were analyzed. Data is presented as a heatmap, with values displayed as relative within each row. Statistical significance was accepted at p<0.05 and is indicated by * (p<0.05), ** (p<0.01), *** (p<0.001) and **** (p<0.0001).

**Figure 3:**
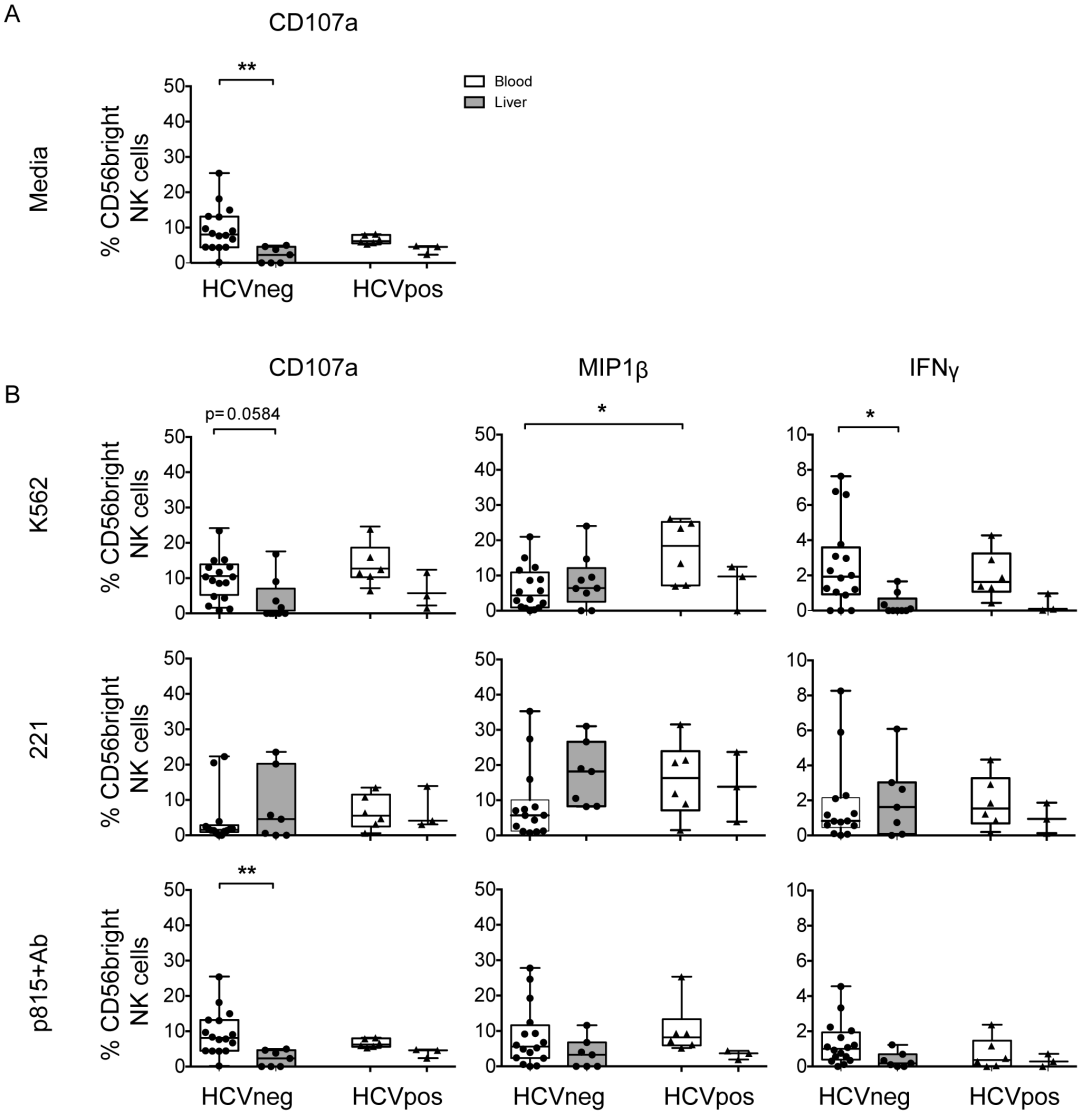
Tissue- and disease-specific differences in NK cell functionality. Functional responses of liver-resident and blood NK cells were assessed in groups of HCV-infected and –uninfected individuals. NK cell degranulation (CD107a) and cytokine production (MIP1β and IFNγ) were measured by flow cytometry in the absence (A) or presence (B) of K562 cells (NKG2D ligation), 721.221 cells (NCR ligation) or antibody-coated p815 cells (ADCC-mediated stimulation). Statistical significance was accepted at p<0.05 and is indicated by * (p<0.05), ** (p<0.01), *** (p<0.001) and **** (p<0.0001).

NK cell activation is governed by a complex interplay of activating and inhibitory receptors [8], [24] that may be perturbed in the context of disease and tissue location [15], [16], [18], [23]. Here we observed an enrichment of NKG2D+, 2B4+ and CD160+ (97.4% vs. 85.65%, p = 0.0011; 90.2% vs. 66.4%, p = 0.008; 90.2% vs. 17.8%, p<0.0001) CD56^bright^ NK cells in the livers of HCV-uninfected, and 2B4+ and CD160+ in HCV-infected individuals (82.75% vs. 61.25%, p = 0.07; 88% versus 22%, p = 0.015) versus the blood compartment (Figure 2A, Figures S3, S4). We also observed a lower frequency of liver-resident NK cells expressing NKp46 and NKp30 in both HCV-infected (54.55% vs. 91.4%, p = 0.0063; 82.15% vs. 96.45%, p = 0.0058) and -uninfected individuals (69.7% vs. 93.4%, p<0.0001; 76.3% vs. 95.7%, p<0.0001, Figure 2A, S1), a phenotype previously associated with recent NK cell activation [13], [25]. Consistent with this, there was a trend towards increased levels of spontaneous degranulation (CD107a), another indicator of NK activation [16], in the liver compared to the blood of HCV-infected individuals, which reached statistical significance in HCV-uninfected individuals (20.3% vs 1.85%, p = 0.0056, Figure 3A). Additionally, fewer NK cells expressed the inhibitory receptor NKG2A, and the ADCC-mediating receptor CD16 in the liver than in the blood in both HCV-infected (73.15% vs. 83.08%, p = 0.025; 10.2% versus 33.45%, p = 0.0055; Figure 2A) and -uninfected individuals (73.15% vs. 89.7%, p = 0.01: 9.9% versus 27.3%, p = 0.0005; Figure 2A). Interestingly, there was also an enrichment of CD161+ cells in the liver-resident CD56^bright^ NK cell subset of HCV-infected and -uninfected individuals (85.85% vs. 41.9%, p<0.0001; 95.2% vs. 65.5%, p<0.0001, Figure 2A). Thus liver-resident CD56^bright^ NK cells exhibit an NCR^low^, CD16^low^, CD107a^high^, NKG2D^high^, 2B4^high^, CD160^high^, CD161^high^, and NKG2A^low^ phenotype, suggesting that NK cells are activated in the liver, and surprisingly were not altered in the context of chronic HCV infection.

### HCV-related liver differences are independent of cirrhosis and liver inflammation

We next asked whether the presence of ongoing inflammation or cirrhosis in the liver might be a confounder in detecting HCV-associated NK cell changes. After stratifying for the presence of cirrhosis, independent of HCV infection, we observed no differences in the NK cell profile between these two groups (Figure S1). Furthermore, the presence of ongoing inflammation was not associated with altered frequencies of NK cell subsets, or the majority of NK cell surface markers (Figure S2A, B). While inflammation did impact the percentage of NK and T cells of total lymphocytes and the frequency of CD56bright NK cells expressing NKp30, exclusion of inflamed livers from the analysis did not alter the HCV-associated profiles described here (Figure S2A, C, D), suggesting that cirrhosis and liver inflammation were not confounding factors in our study.

### Liver NK cells have reduced cytotoxicity and ADCC function compared to circulating NK cells

Given the limited differences observed in NK cell immunophenotypes in the livers of HCV-infected and uninfected patients, we next sought to define whether liver-resident CD56^bright^ NK cells were functionally distinct from those in the blood in HCV infection. NK cell function was assessed against a panel of target cells including an NKG2D-target cell line (K562), NCR-target cell line (721.221) and an ADCC-target cell line (antibody-coated p815 cells). Overall, functional responses of liver-resident CD56^bright^ NK cells were distinct from those of the blood in both HCV-infected and -uninfected individuals (Figure 3B). Specifically, in uninfected individuals, compared to the blood, liver-resident CD56^bright^ NK cells exhibited reduced degranulation following stimulation with ADCC and NKG2D targets (4.61% vs. 6.24%, p = 0.0032; 0% vs. 9.83%, p = 0.0584) and impaired IFNγ production following stimulation with NKG2D targets (0.01% vs. 1.63%, p = 0.014), while MIP1B production was maintained in response to all stimuli. ADCC-mediated responses were consistent with the expression of CD16 on CD56^bright^ NK cells the frequency of which significantly correlated with ADCC-mediated degranulation and MIP1β production in the blood (Figure S5). When we compared blood NK cells from HCV-infected and –uninfected individuals we observed greater production of MIP1β following NKG2D-mediated stimulation in HCV-infected individuals (18.38% vs. 4.32%, p = 0.045). The function of CD56dim NK cells showed similar patterns (Figure S6). Thus, overall, liver NK cells appear less functionally active than blood NK cells upon stimulation, and consistent with the unaltered immunophenotype observed here (Fig 2), remained unperturbed in the context of HCV infection, while the ability of blood NK cells to selectively produce the chemo-attractant MIP1β was enhanced with HCV infection.

### High levels of NKp46 and NKG2D on CD56^bright^ liver NK cells is associated with low fibrosis

**Figure 4:**
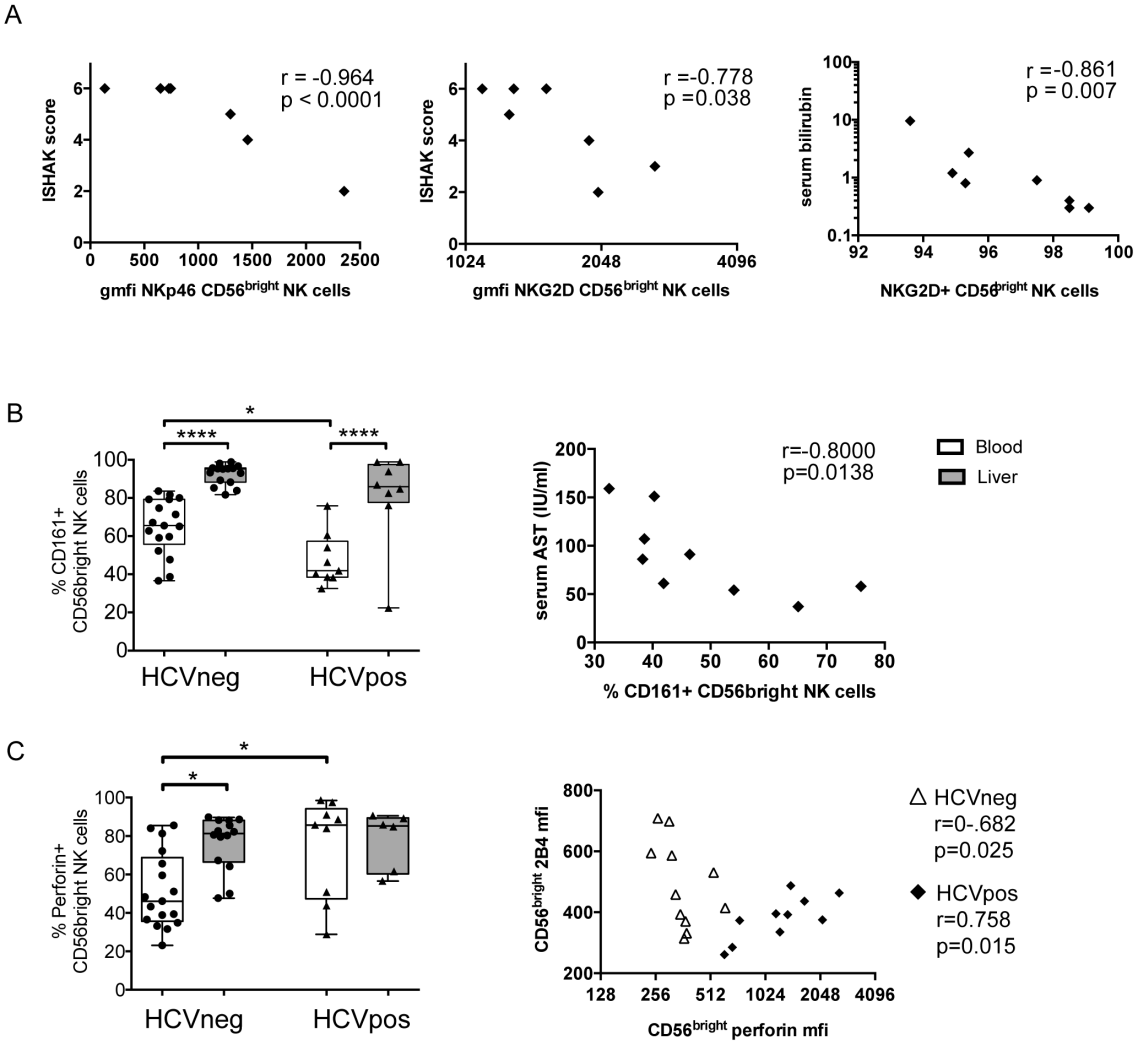
Unique liver and blood NK phenotypes associate with markers of disease progression. Correlative analysis of NK cell immunophenotype and function in the liver and blood with clinical measures of HCV infection such as ISHAK score, ALT, AST, serum bilirubin, HCV RNA and INR were performed. The significant associations of liver NK cell receptors with clinical markers are displayed (A) along with correlations between the frequency of CD161+, (B) and perforin expression on blood CD56^bright^ NK cells (C) with clinical markers and the expression of other NK cell receptors. Correlations were done using Spearman's rank test and statistical significance was accepted at p<0.05 and is indicated by * (p<0.05), ** (p<0.01) and *** (p<0.001).

Despite observing limited alterations in the liver NK cell immunophenotype and function in HCV infection, we investigated whether a particular NK cell profile was linked to liver damage, given that chronic HCV is associated with increased liver fibrosis and incidence of cancer. We observed that CD56^bright^ liver NK cells expressing high levels of the activating receptors NKp46 and NKG2D were inversely correlated with ISHAK score, a clinical measure of liver fibrosis (r = −0.9684, p<0.0001; r = −0.778, p = 0.038; figure 4A), and that the frequency of NKG2D+ CD56^bright^ NK cells in the liver was inversely correlated with serum bilirubin (r = −0.861, p = 0.007; Figure 4A) which is a marker of liver function, cholestasis and hemoglobin turnover. These data suggest that NK cells are associated with altered liver function and may play a role in modulating liver fibrosis in chronic HCV infection.

### HCV infection is linked to higher perforin expression and lower frequency of CD161+ blood NK cells

Discrete NK cell patterns in the blood have previously been reported in chronic HCV infection [14]. We therefore investigated whether blood NK cells displayed a distinct HCV-specific profile in our cohort. We observed a lower frequency of NK cells expressing the inhibitory receptor CD161, and an enrichment of perforin-expressing CD56^bright^ NK cells in the blood of HCV-infected versus -uninfected individuals (41.9% vs. 65.5%, p = 0.012; 85.8% vs. 46.1%, p = 0.017, Figure 4B). This was accompanied by higher expression of perforin on a per cell basis (p = 0.014, data not shown), indicating that not only are there more perforin-expressing NK cells in the blood of HCV-infected individuals but that each of these cells contains more perforin. No other differences in NK cell markers were observed in the blood between HCV-infected and –uninfected individuals (Figure 2B). This suggests that the inhibitory profile of blood NK cells and the regulation of perforin may be altered in the context of chronic HCV infection, and may result in a more cytolytic NK cell profile following chronic exposure to inflammatory or viral signals.

### Perforin^high^CD161^low^ blood NK cells are linked to liver inflammation and altered activation profiles

In light of the clear enrichment of perforin^high^CD161^low^ cells in the blood of HCV-infected individuals, we next aimed to characterize the relationship between this population of NK cells in HCV-infected subjects, and markers of disease pathology. Interestingly, the frequency of CD161+ CD56^bright^ NK cells in the blood was inversely correlated with serum levels of aspartate aminotransferase (AST, r = −0.8, p = 0.0138, Figure 4B), a liver enzyme associated with acute liver damage, suggesting there may be an association of these cells with liver inflammation. Moreover, expression of perforin was associated with elevated 2B4 expression (r = 0.75, p = 0.0149) in the CD56^bright^ NK cell subset (Figure 4C). In contrast to HCV infected individuals, perforin expression was inversely correlated with 2B4 expression (r = −0.68, p = 0.0251) suggesting that HCV infection may significantly alter the relationship between these molecules, resulting in the expansion of a unique population of NK cells in the blood marked by perforin^high^CD161^low^2B4^high^CD56^bright^.

## Discussion

Mounting evidence supports a role for NK cells in antiviral control of HCV [10], [13], [26]–[29], yet the majority of studies to date have focused on the activity of blood NK cells, and only a few studies have probed the NK cell response at the site of infection, the liver. Furthermore, previous reports published on the properties of intrahepatic NK cells are conflicting, likely related to limited cell numbers from biopsies or lack of appropriate controls [15], [16], [30], [31]. Therefore, here we aimed to carefully characterize blood and liver NK cells from both HCV-infected and -uninfected donors from whom intrahepatic cells were obtained following liver surgery (Tables 1, 2). We observed that the liver, like other organs [18] was enriched for CD56^bright^ NK cells, which exhibited an activated profile, marked by NCR^low^NKG2A^low^CD107a^high^CD160^high^NKG2D^high^ and displayed suppressed ADCC- and NKG2D-mediated recognition of target cells. Interestingly, the liver-resident NK cell immunophenotype appeared unperturbed in the context of chronic HCV infection, whereas more striking differences were observed in the blood, marked by the expansion of a CD161^low^perforin^high^ NK cell population. These data surprisingly show NK cell phenotypes and functions in the liver that are relatively preserved in the presence of chronic HCV infection, while a stronger impact is observed in circulating NK cells.

A key difference between liver-resident NK cells and those from the blood in all cases, irrespective of liver pathology, was reduced NCR expression. Activation of NK cells results in rapid downregulation of NKp46 and NKp30 through the action of matrix metalloproteinase [13], [25], [32], possibly as a mechanism of avoiding activation-induced cell death, and therefore represents a potential marker of recent activation. Furthermore, liver NK cells exhibited dampened NKG2D- and ADCC-mediated antiviral function while production of the chemoattractant cytokine MIP1β was maintained, independent of infection, demonstrating selective inhibition of cytolytic NK cell activity within this tissue. However, the liver appears to be a naturally immunosuppressed environment [33] requiring specific signals, such as interferons [34], to elicit cytolytic responses in the presence of classic activating signals [35]. Thus, functional differences between liver and blood NK cells should perhaps be expected, as the suppression of NK cell responses through specific pathways may be important in preventing excessive liver damage during infection, while chemokine secretion may permit the effective or enhanced recruitment of other effector cells to the sites of infection to effectively control viral replication. Thus, while the studies presented here were performed on limited numbers of samples, they provide evidence for a distinct functional signature of NK cells in the liver, which may warrant future studies on larger patient populations.

Interestingly, the profile of intrahepatic NK cells was remarkably unaltered in chronic HCV infection. This was in contrast to previous reports demonstrating distinct NK cell profiles in blood [14] and liver [15], [16]. Specifically, Nattermann et al (2006) demonstrated that differences could be successfully identified in a relatively small cohort. However, sufficient statistical power existed in the study detailed here to resolve significant differences in NK cell phenotypic differences, and lack of concordance with previous studies likely reflects differences in biopsy collection from subjects at divergent stages of disease. Further studies using larger patient cohorts may however provide greater opportunity in the identification of more subtle differences in NK cell phenotype and function in the liver at different stages of the disease.

In our cohort we observed that high levels of the activating receptors NKp46 and NKG2D tracked with lower liver fibrosis and function. This has previously been shown for NKp46 [36] linked to efficient NK cell mediated killing of stellate cells [37], suggesting that the enrichment of these cells in the liver may help control fibrosis. Moreover, the relative lack of NK cell perturbation in the liver in chronic HCV infection may not be surprising given that NK cells are typically involved in the initial innate immune response to acute viral infections. As such, it is plausible that studies on liver samples from chronically infected subjects may have missed the window of time when the most robust waves of NK cell responses may be elicited. Therefore, in sampling during chronic infection, we may be looking at a cell population geared towards limiting fibrosis rather than viral suppression. Thus, future studies should focus on acutely infected subjects that either go on to resolve infection or develop chronic HCV disease. This should be feasible through the employment of elective fine needle aspirates from the liver [38], [39] and should provide insights into the nature of NK cell responses that do or do not control viremia.

In contrast to the liver, we observed a distinct NK cell profile in the blood that tracked with HCV infection and was characterized by low frequencies of CD161+ cells and high levels of perforin in the CD56^bright^ population. Reduced frequencies of CD161+ NK cells have previously been observed in chronic HCV and HIV infection [13], [28] suggesting that cells with this specific phenotype respond to HCV infection. Interestingly, we found an inverse relationship between the frequency of CD161+ CD56^bright^ NK cells and levels of serum AST in our cohort, suggesting a link between this marker and increased inflammation in the liver. As such, liver inflammation could drive increased homing of CD161+ NK cells to the liver. However, we observed no increase in the frequency of CD161+ NK cells in the liver in HCV infection, raising the question whether these cells might ultimately get deleted in high-level viremia that persists for many years. Alternatively, long-term exposure to inflammatory signals may result in downregulation of the inhibitory CD161 receptor on CD56^bright^ NK cells, resulting in a more activated NK cell population in chronic disease. Moreover, elevated perforin expression in CD56^bright^ NK cells in HCV infection was associated with a distinct 2B4^high^ immunophenotype. Perforin is an important cytolytic molecule and its elevation in chronic HCV infection may suggest increased cytolytic potency of blood NK cells, potentially driven by chronic inflammatory signals. Consequently, perforin-mediated mechanisms of NK cell killing may be boosted in chronic HCV infection. Interestingly, T cell are major drivers of liver fibrosis in HCV infection [40] and recent evidence has shown that in the setting of chronic infection, perforin-mediated modulation of virus-specific T cell responses by NK cells is crucial in limiting liver damage [17], [41]–[44]. Moreover, the key NK cell receptor involved in this mechanism was 2B4 [17], [45], suggesting that the altered relationship we observed between 2B4 and perforin in chronic HCV infection to be indicative of blood NK cells tuned towards modulation of T cell responses in chronic disease, rather than direct suppression of viral replication, with the goal of limiting immunopathology.

Overall, we demonstrate that tissue localization is the dominant factor influencing NK phenotype and functional profiles. Surprisingly, HCV infection more broadly impacts the profiles of NK cells circulating in the blood, compared to those in the liver. Yet subtle differences in NK cell phenotype in the liver were associated with distinct clinical outcomes, suggesting that in chronic infection, intrahepatic NK cells may be functionally tuned towards modulating immunopathology, rather than limiting viral replication. Additional studies, on liver-resident NK cells during acute infection, a time when innate immune cellular activity may be most relevant, could provide greater insights into the role of this cytolytic lymphocyte subset, which may be a critical component of early antiviral immunity.

## Supporting Information

Figure S1 **Intrahepatic NK cell profiles in cirrhotic livers.** Box plots comparing the frequency of (A) NK and T cells, NK cell subsets and (B) expression of NK cell receptors on resting intrahepatic CD56bright NK cells, as well as K562-stimulated functional responses (CD107a, IFNγ and MIP1β production), stratified by the presence or absence of cirrhosis.
(TIF)

Figure S2 **Intrahepatic NK cell profiles in inflamed livers.** Box plots comparing the frequency of (A) NK and T cells, NK cell subsets and (B) expression of NK cell receptors on resting intrahepatic CD56bright NK cells, stratified by the presence or absence of ongoing liver inflammation. (C) Comparison of NKp30 expression on intrahepatic CD56bright NK cells in the presence or absence of liver inflammation and, following exclusion of inflamed livers, in the presence or absence of HCV infection. (D) Comparison of percentage of NK cells and T cells of total lymphocytes in presence of absence of HCV infection following exclusion of inflamed livers.
(AI) Click here for additional data file.

Figure S3 **Key tissue and disease specific CD56^bright^ NK cell differences.** Scatter plots showing the significant immunophenotypic differences between blood and liver-resident CD56bright NK cell populations in groups of HCV-infected and uninfected individuals. Statistical significance was accepted at p<0.05 and is indicated by * (p<0.05), ** (p<0.01) and *** (p<0.001).
(TIF)

Figure S4 **Representative FACS plots of phenotypic NK cell receptors.** Representative flow cytometry plots showing the key immunophenotypic differences between blood and liver-resident CD56bright NK cell populations in groups of HCV-infected and uninfected individuals.
(TIF)

Figure S5 **CD16 expression on CD56^bright^ NK cells and ADCC-mediated functionality.** Representative flow cytometry plots show gating on the CD56^bright^ population and identification of the CD16+ subset (A). Following stimulation with antibody-coated p815 cells, the function of CD56bright NK cells was assessed by flow cytometry (B). Baseline expression of CD16 on the CD56^bright^ population in the liver and blood of HCV-infected and uninfected patients was assessed (C). CD56^bright^ NK cell function correlated with the frequency of CD16+ cells (D). Statistical significance was accepted at p<0.05 and is indicated by * (p<0.05), ** (p<0.01), *** (p<0.001) and **** (p<0.0001).
(TIF)

Figure S6 **Tissue- and disease-specific differences in CD56^dim^ NK cell functionality.** Functional responses of liver-resident and blood CD56^dim^ NK cells were assessed in groups of HCV-infected and –uninfected individuals. NK cell degranulation (CD107a) and cytokine production (MIP1β and IFNγ) were measured by flow cytometry in the absence (A) or presence (B) of K562 cells (NKG2D ligation), 721.221 cells (NCR ligation) or antibody-coated p815 cells (ADCC-mediated stimulation). Statistical significance was accepted at p<0.05 and is indicated by * (p<0.05), ** (p<0.01), *** (p<0.001) and **** (p<0.0001).
(TIF)

## References

[1] Ghany MG, Strader DB, Thomas DL, Seeff LB (2009) American Association for the Study of Liver Diseases (2009) Diagnosis, management, and treatment of hepatitis C: an update. Hepatology 49: 1335–1374 10.1002/hep.22759 19330875 PMC7477893

[2] Lauer GM (2013) Immune responses to hepatitis C virus (HCV) infection and the prospects for an effective HCV vaccine or immunotherapies. J Infect Dis 207 Suppl 1S7–S12 10.1093/infdis/jis762 23390305

[3] Liang TJ (2013) Current progress in development of hepatitis C virus vaccines. Nat Med 19: 869–878 10.1038/nm.3183 23836237 PMC6263146

[4] Houghton M (2011) Prospects for prophylactic and therapeutic vaccines against the hepatitis C viruses. Immunol Rev 239: 99–108 10.1111/j.1600-065X.2010.00977.x 21198667

[5] Carrega P, Ferlazzo G (2012) Natural killer cell distribution and trafficking in human tissues. Front Immunol 3: 347 10.3389/fimmu.2012.00347 23230434 PMC3515878

[6] Cooper MA, Fehniger TA, Caligiuri MA (2001) The biology of human natural killer-cell subsets. Trends Immunol 22: 633–640 10.1016/S1471-4906(01)02060-9 11698225

[7] Caligiuri MA (2008) Human natural killer cells. Blood 112: 461–469 10.1182/blood-2007-09-077438 18650461 PMC2481557

[8] Lanier L (2005) NK cell recognition. Annu Rev Immunol 23: 225–274. 15771571 10.1146/annurev.immunol.23.021704.115526

[9] Klugewitz K, Adams DH, Emoto M, Eulenburg K, Hamann A (2004) The composition of intrahepatic lymphocytes: shaped by selective recruitment? Trends Immunol 25: 590–594 10.1016/j.it.2004.09.006 15489187

[10] Khakoo SI, Thio CL, Martin MP, Brooks CR, Gao X, et al (2004) HLA and NK Cell Inhibitory Receptor Genes in Resolving Hepatitis C Virus Infection. Science Signaling 305: 872–787 10.1126/science.1097670 15297676

[11] Knapp S, Warshow U, Hegazy D, Brackenbury L, Guha IN, et al (2010) Consistent beneficial effects of killer cell immunoglobulin-like receptor 2DL3 and group 1 human leukocyte antigen-C following exposure to hepatitis C virus. Hepatology 51: 1168–1175 10.1002/hep.23477 20077564 PMC4202114

[12] Romero V, Azocar J, Zúñiga J, Clavijo OP, Terreros D, et al (2008) Interaction of NK inhibitory receptor genes with HLA-C and MHC class II alleles in Hepatitis C virus infection outcome. Mol Immunol 45: 2429–2436 10.1016/j.molimm.2008.01.002 18289678 PMC2387047

[13] Alter G, Jost S, Rihn S, Reyor LL, Nolan BE, et al (2011) Reduced frequencies of NKp30+NKp46+, CD161+, and NKG2D+ NK cells in acute HCV infection may predict viral clearance. Journal of Hepatology 55: 278–288 10.1016/j.jhep.2010.11.030 21168454 PMC3729214

[14] Oliviero B, Varchetta S, Paudice E, Michelone G, Zaramella M, et al (2009) Natural killer cell functional dichotomy in chronic hepatitis B and chronic hepatitis C virus infections. Gastroenterology 137: 1151–60–1160.e1–7 10.1053/j.gastro.2009.05.047 19470388

[15] Nattermann J, Feldmann G, Ahlenstiel G, Langhans B, Sauerbruch T, et al. (2006) Surface expression and cytolytic function of natural killer cell receptors is altered in chronic hepatitis C. Gut. Available: http://gut.bmj.com/content/55/6/869.abstract 10.1136/gut.2005.076463 PMC1856237 16322112

[16] Varchetta S, Mele D, Mantovani S, Oliviero B, Cremonesi E, et al.. (2012) Impaired intrahepatic natural killer cell cytotoxic function in chronic hepatitis C virus infection. Hepatology: n/a–n/a. doi:10.1002/hep.25723 10.1002/hep.25723 22431186

[17] Waggoner SN, Cornberg M, Selin LK, Welsh RM (2012) Natural killer cells act as rheostats modulating antiviral T cells. Nature 481: 394–398 10.1038/nature10624 PMC3539796 22101430

[18] Ferlazzo G, Thomas D, Lin S-L, Goodman K, Morandi B, et al (2004) The abundant NK cells in human secondary lymphoid tissues require activation to express killer cell Ig-like receptors and become cytolytic. J Immunol 172: 1455–1462. 14734722 10.4049/jimmunol.172.3.1455

[19] Kroy DC, Ciuffreda D, Cooperrider JH, Tomlinson M, Hauck GD, et al (2014) Liver Environment and HCV Replication Affect Human T-Cell Phenotype and Expression of Inhibitory Receptors. Gastroenterology 146: 550–561 10.1053/j.gastro.2013.10.022 24148617 PMC3946973

[20] Doherty DG, O'Farrelly C (2000) Innate and adaptive lymphoid cells in the human liver. Immunol Rev 174: 5–20. 10807503 10.1034/j.1600-0528.2002.017416.x

[21] Gao B, Radaeva S, Park O (2009) Liver natural killer and natural killer T cells: immunobiology and emerging roles in liver diseases. J Leukoc Biol 86: 513–528 10.1189/JLB.0309135 19542050 PMC2735282

[22] Kawarabayashi N, Seki S, Hatsuse K (2000) Decrease of CD56+T cells and natural killer cells in cirrhotic livers with hepatitis C may be involved in their susceptibility to hepatocellular carcinoma. Hepatology 32: 962–969 10.1053/jhep.2000.19362 11050046 PMC7165992

[23] Burt BM, Plitas G, Zhao Z, Bamboat ZM, Nguyen HM, et al (2009) The lytic potential of human liver NK cells is restricted by their limited expression of inhibitory killer Ig-like receptors. The Journal of Immunology 183: 1789–1796 10.4049/jimmunol.0900541 19587011 PMC3253491

[24] Bryceson YT, March ME, Ljunggren H-G, Long EO (2006) Synergy among receptors on resting NK cells for the activation of natural cytotoxicity and cytokine secretion. Blood 107: 159–166 10.1182/blood-2005-04-1351 16150947 PMC1895346

[25] Fogli M, Costa P, Murdaca G, Setti M, Mingari MC, et al (2004) Significant NK cell activation associated with decreased cytolytic function in peripheral blood of HIV-1-infected patients. Eur J Immunol 34: 2313–2321 10.1002/eji.200425251 15259029

[26] Ahlenstiel G, Edlich B, Hogdal LJ, Rotman Y, Noureddin M, et al (2011) Early changes in natural killer cell function indicate virologic response to interferon therapy for hepatitis C. Gastroenterology 141: 1231–9–1239.e1–2 10.1053/j.gastro.2011.06.069 21741920 PMC3353552

[27] Farag MMS, Weigand K, Encke J, Momburg F (2011) Activation of natural killer cells by hepatitis C virus particles in vitro. Clin Exp Immunol 165: 352–362 10.1111/j.1365-2249.2011.04431.x 21682720 PMC3170984

[28] Pelletier S, Drouin C, Bédard N, Khakoo SI, Bruneau J, et al (2010) Increased degranulation of natural killer cells during acute HCV correlates with the magnitude of virus-specific T cell responses. Journal of Hepatology 53: 805–816 10.1016/j.jhep.2010.05.013 20688412 PMC4178223

[29] Stegmann KA, Björkström NK, Veber H, Ciesek S, Riese P, et al (2010) Interferon-alpha-induced TRAIL on natural killer cells is associated with control of hepatitis C virus infection. Gastroenterology 138: 1885–1897 10.1053/j.gastro.2010.01.051 20334827

[30] Bonorino P, Ramzan M, Camous X, Dufeu-Duchesne T, Thélu M-A, et al (2009) Fine characterization of intrahepatic NK cells expressing natural killer receptors in chronic hepatitis B and C. Journal of Hepatology 51: 458–467 10.1016/j.jhep.2009.05.030 19596474

[31] Yamagiwa S, Matsuda Y, Ichida T, Honda Y, Takamura M, et al (2008) Sustained response to interferon-alpha plus ribavirin therapy for chronic hepatitis C is closely associated with increased dynamism of intrahepatic natural killer and natural killer T cells. Hepatol Res 38: 664–672 10.1111/j.1872-034X.2008.00317.x 18328072

[32] Parsons MS, Tang C-C, Jegaskanda S (2014) Center RJ, Brooks AG, et al (2014) Anti-HIV antibody-dependent activation of NK cells impairs NKp46 expression. The Journal of Immunology 192: 308–315 10.4049/jimmunol.1301247 24319263

[33] Knolle PA, Gerken G (2000) Local control of the immune response in the liver. Immunol Rev 174: 21–34. 10807504 10.1034/j.1600-0528.2002.017408.x

[34] Lang PA, Recher M, Honke N, Scheu S, Borkens S, et al (2010) Tissue macrophages suppress viral replication and prevent severe immunopathology in an interferon-I-dependent manner in mice. Hepatology 52: 25–32 10.1002/hep.23640 20578253

[35] Yang Y, Xiang Z, Ertl HC, Wilson JM (1995) Upregulation of class I major histocompatibility complex antigens by interferon gamma is necessary for T-cell-mediated elimination of recombinant adenovirus-infected hepatocytes in vivo. Proc Natl Acad Sci USA 92: 7257–7261. 7638177 10.1073/pnas.92.16.7257 PMC41318

[36] Krämer B, Körner C, Kebschull M, Glässner A, Eisenhardt M, et al (2012) Natural killer p46(High) expression defines a natural killer cell subset that is potentially involved in control of hepatitis C virus replication and modulation of liver fibrosis. Hepatology 56: 1201–1213 10.1002/hep.25804 22532190

[37] Glässner A, Eisenhardt M, Krämer B, Körner C, Coenen M, et al (2012) NK cells from HCV-infected patients effectively induce apoptosis of activated primary human hepatic stellate cells in a TRAIL-, FasL- and NKG2D-dependent manner. Lab Invest 92: 967–977 10.1038/labinvest.2012.54 22449797

[38] Pembroke T, Christian A, Jones E, Hills RK, Wang ECY, et al (2014) The paradox of NKp46+ natural killer cells: drivers of severe hepatitis C virus-induced pathology but in-vivo resistance to interferon α treatment. Gut 63: 515–524 10.1136/gutjnl-2013-304472 23665989 PMC3932740

[39] Khashab M, Mokadem M, DeWitt J, Emerson R, Sherman S, et al (2010) Endoscopic ultrasound-guided fine-needle aspiration with or without flow cytometry for the diagnosis of primary pancreatic lymphoma – a case series. Endoscopy 42: 228–231 10.1055/s-0029-1243859 20101569

[40] Golden-Mason L, Rosen HR (2013) Natural killer cells: multifaceted players with key roles in hepatitis C immunity. Immunol Rev 255: 68–81 10.1111/imr.12090 23947348 PMC3765000

[41] Lang PA, Lang KS, Xu HC, Grusdat M, Parish IA, et al (2012) Natural killer cell activation enhances immune pathology and promotes chronic infection by limiting CD8+ T-cell immunity. Proceedings of the National Academy of Sciences 109: 1210–1215 10.1073/pnas.1118834109 PMC3268324 22167808

[42] Stepp SE, Mathew PA, Bennett M, de Saint Basile G, Kumar V (2000) Perforin: more than just an effector molecule. Immunology Today 21: 254–256 10.1016/S0167-5699(00)01622-4 10825735

[43] Matloubian M, Suresh M, Glass A, Galvan M, Chow K, et al (1999) A role for perforin in downregulating T-cell responses during chronic viral infection. Journal of Virology 73: 2527–2536. 9971838 10.1128/jvi.73.3.2527-2536.1999 PMC104500

[44] van Dommelen SLH, Sumaria N, Schreiber RD, Scalzo AA, Smyth MJ, et al (2006) Perforin and granzymes have distinct roles in defensive immunity and immunopathology. Immunity 25: 835–848 10.1016/j.immuni.2006.09.010 17088087

[45] Waggoner SN, Taniguchi RT, Mathew PA, Kumar V, Welsh RM (2010) Absence of mouse 2B4 promotes NK cell-mediated killing of activated CD8+ T cells, leading to prolonged viral persistence and altered pathogenesis. J Clin Invest 120: 1925–1938 10.1172/JCI41264 20440077 PMC2877945

