# Supplementary figures and images for: Chronic HCV Infection Affects the NK Cell Phenotype in the Blood More than in the Liver
Source: PLoS One. 2014 Aug 22;9(8):e105950. doi: 10.1371/journal.pone.0105950 (PMC4141847; doi:10.1371/journal.pone.0105950)

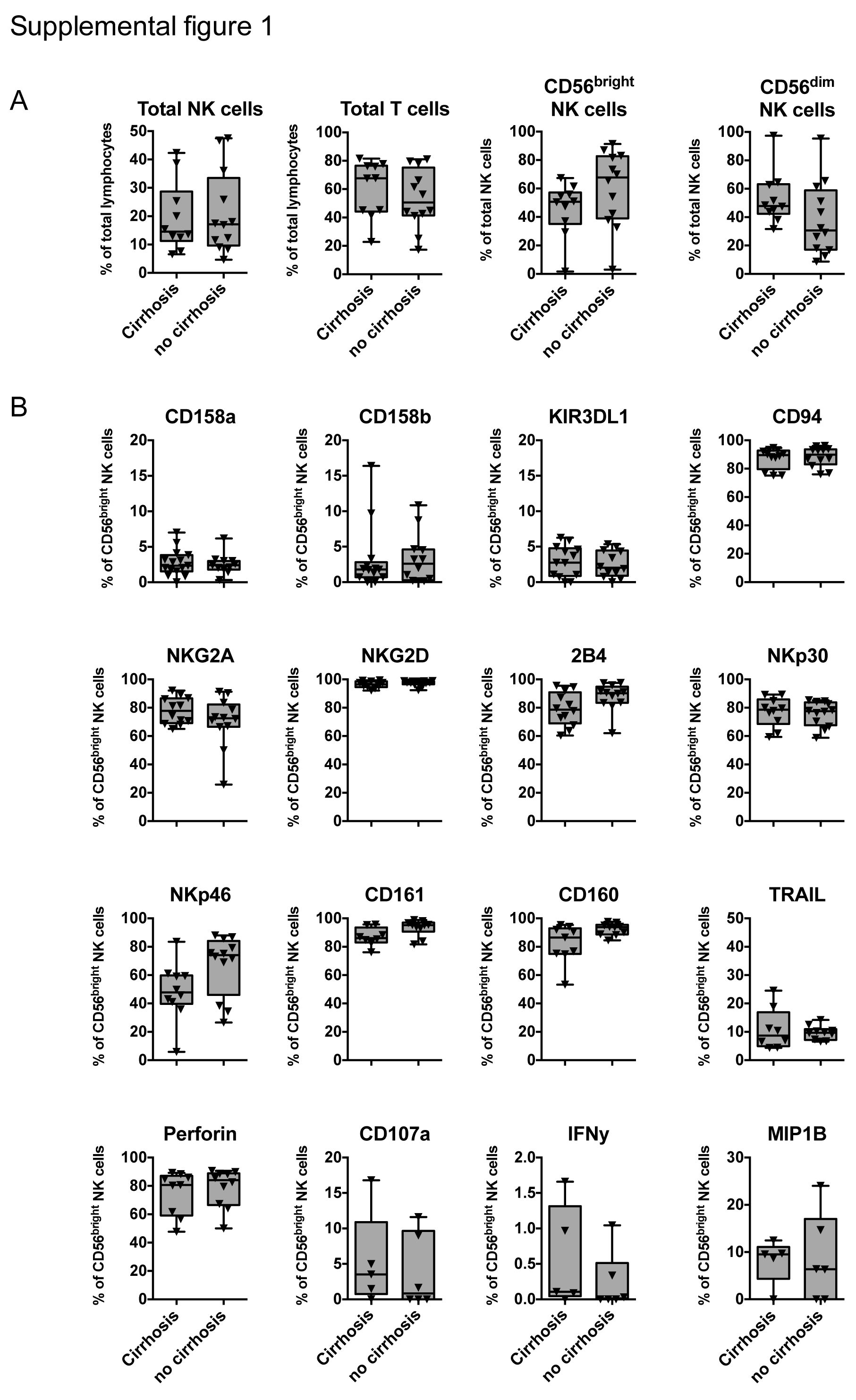

Supplement: Figure S1 — Intrahepatic NK cell profiles in cirrhotic livers. Box plots comparing the frequency of (A) NK and T cells, NK cell subsets and (B) expression of NK cell receptors on resting intrahepatic CD56bright NK cells, as well as K562-stimulated functional responses (CD107a, IFNγ and MIP1β production), stratified by the presence or absence of cirrhosis. (TIF) [file pone.0105950.s001.tif]

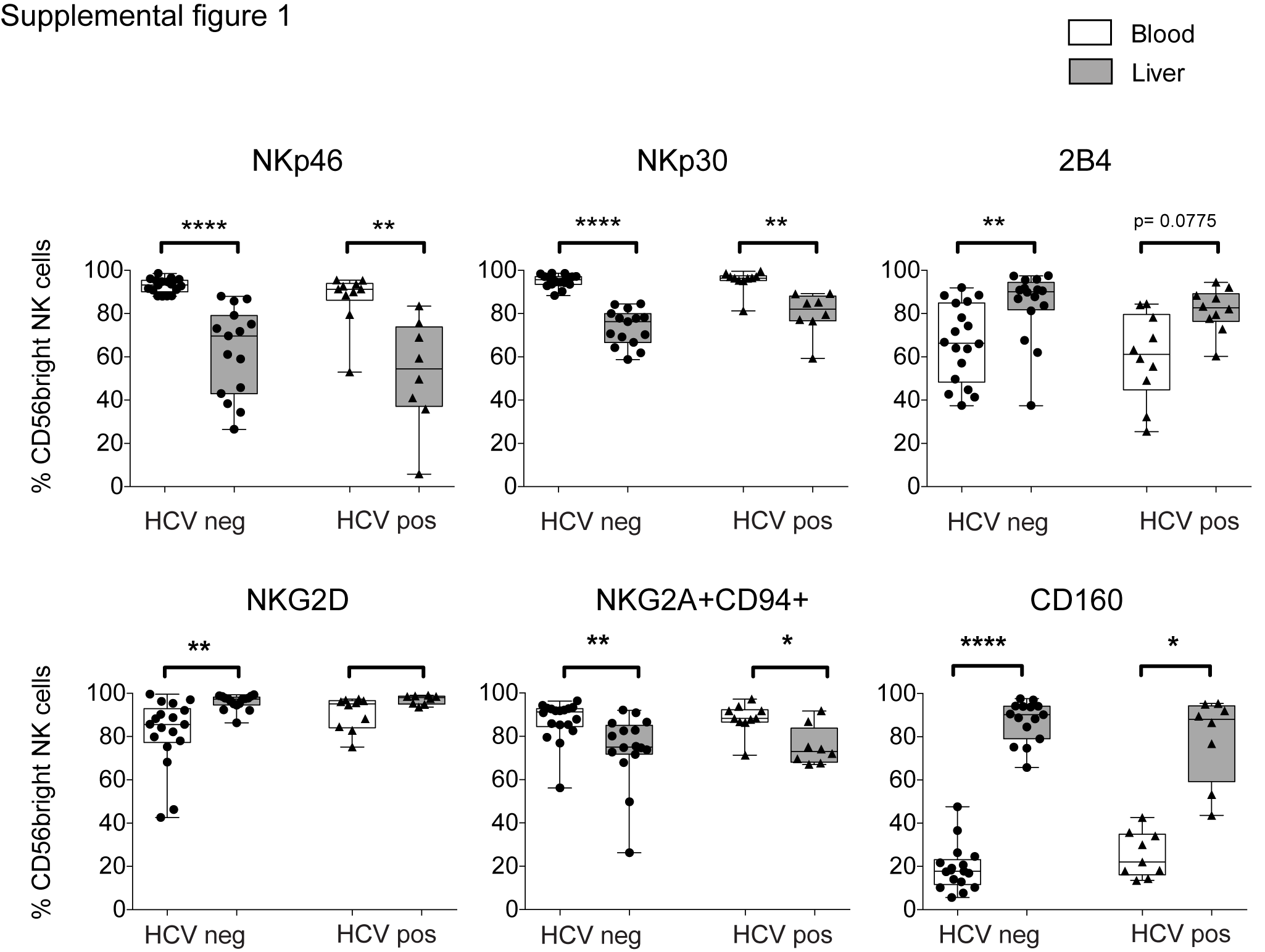

Supplement: Figure S3 — Key tissue and disease specific CD56bright NK cell differences. Scatter plots showing the significant immunophenotypic differences between blood and liver-resident CD56bright NK cell populations in groups of HCV-infected and uninfected individuals. Statistical significance was accepted at p<0.05 and is indicated by * (p<0.05), ** (p<0.01) and *** (p<0.001). (TIF) [file pone.0105950.s003.tif]

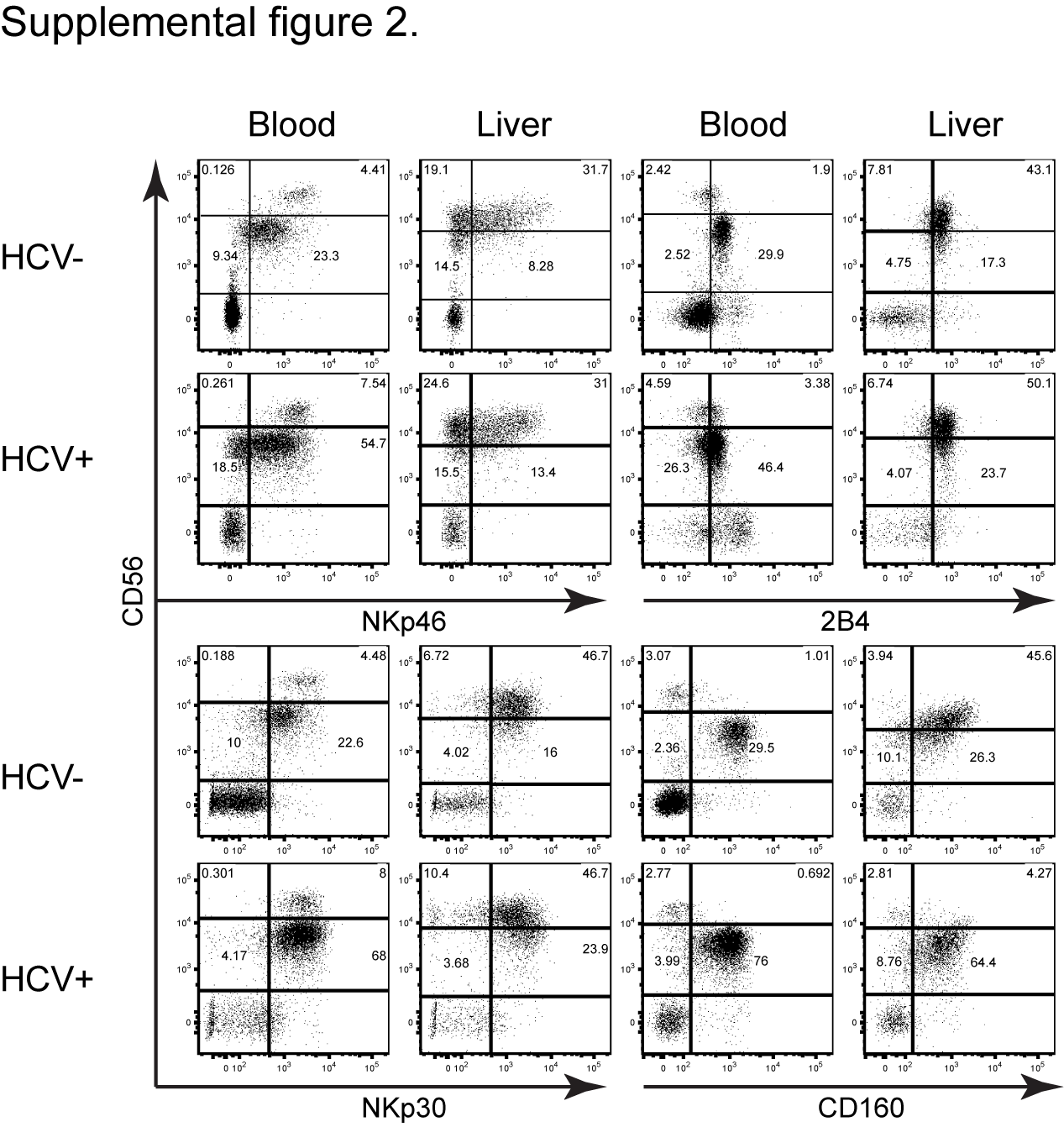

Supplement: Figure S4 — Representative FACS plots of phenotypic NK cell receptors. Representative flow cytometry plots showing the key immunophenotypic differences between blood and liver-resident CD56bright NK cell populations in groups of HCV-infected and uninfected individuals. (TIF) [file pone.0105950.s004.tif]

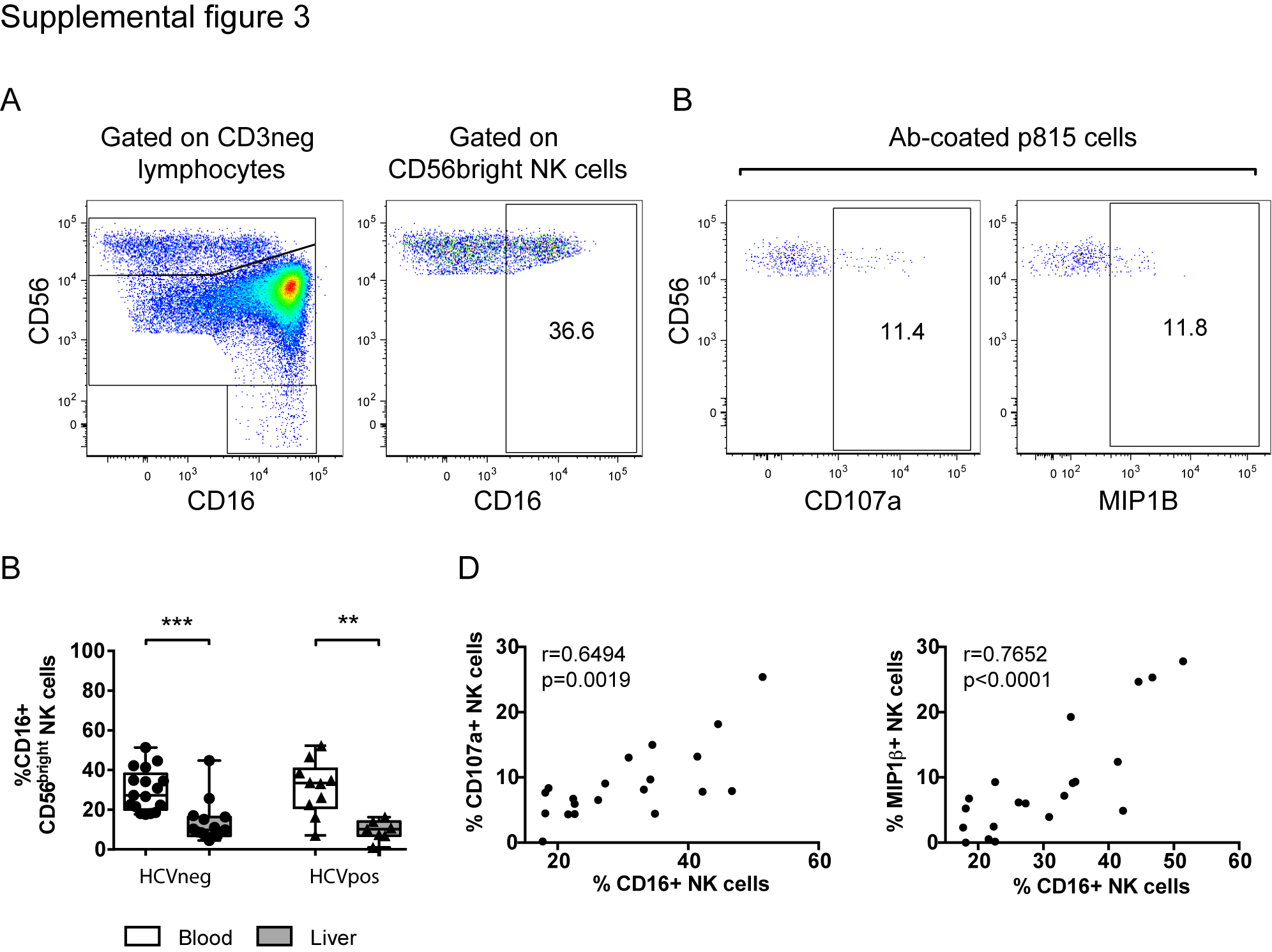

Supplement: Figure S5 — CD16 expression on CD56bright NK cells and ADCC-mediated functionality. Representative flow cytometry plots show gating on the CD56bright population and identification of the CD16+ subset (A). Following stimulation with antibody-coated p815 cells, the function of CD56bright NK cells was assessed by flow cytometry (B). Baseline expression of CD16 on the CD56bright population in the liver and blood of HCV-infected and uninfected patients was assessed (C). CD56bright NK cell function correlated with the frequency of CD16+ cells (D). Statistical significance was accepted at p<0.05 and is indicated by * (p<0.05), ** (p<0.01), *** (p<0.001) and **** (p<0.0001). (TIF) [file pone.0105950.s005.tif]

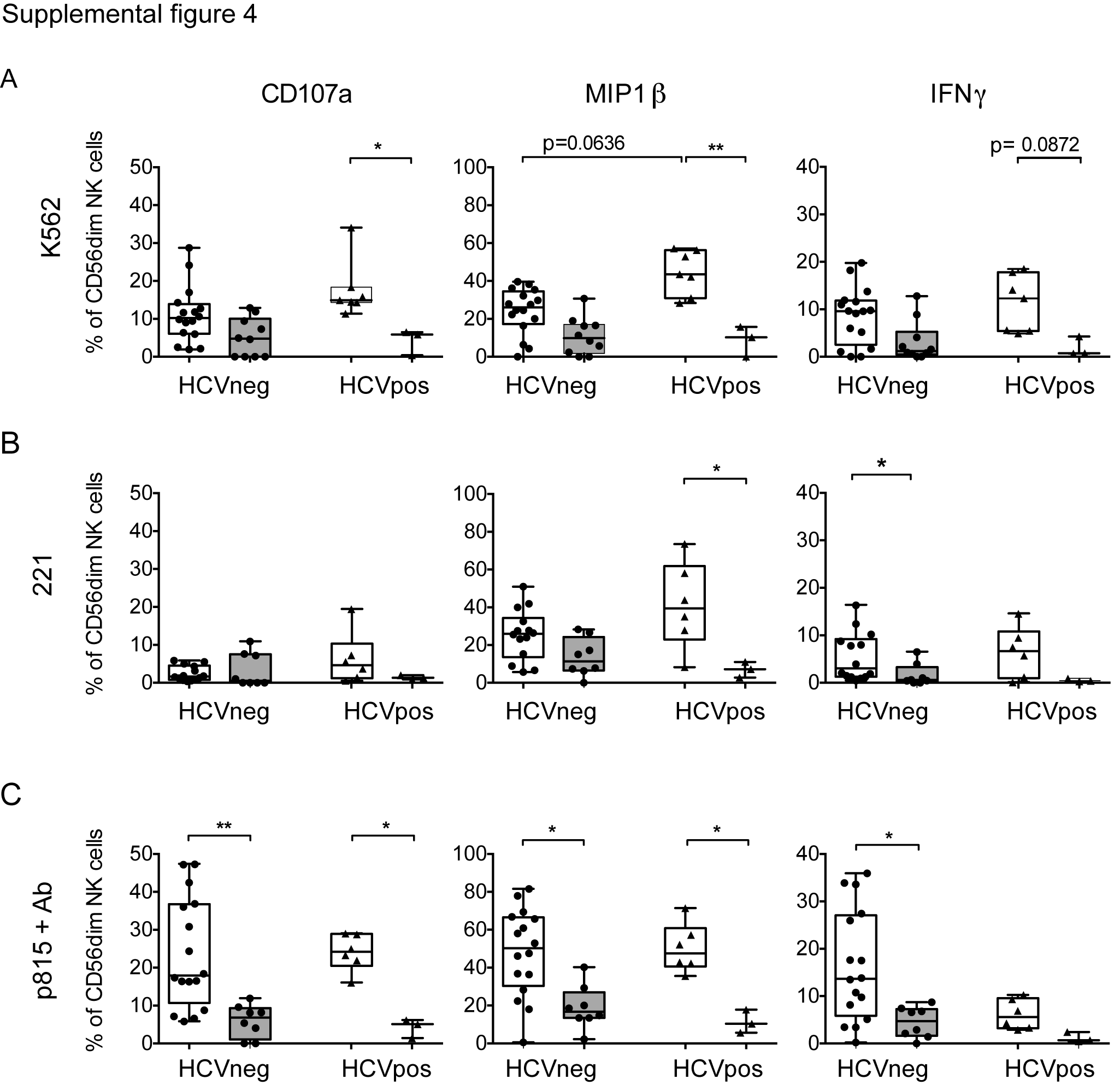

Supplement: Figure S6 — Tissue- and disease-specific differences in CD56dim NK cell functionality. Functional responses of liver-resident and blood CD56dim NK cells were assessed in groups of HCV-infected and –uninfected individuals. NK cell degranulation (CD107a) and cytokine production (MIP1β and IFNγ) were measured by flow cytometry in the absence (A) or presence (B) of K562 cells (NKG2D ligation), 721.221 cells (NCR ligation) or antibody-coated p815 cells (ADCC-mediated stimulation). Statistical significance was accepted at p<0.05 and is indicated by * (p<0.05), ** (p<0.01), *** (p<0.001) and **** (p<0.0001). (TIF) [file pone.0105950.s006.tif]
